# Tracheocele: A Rare Airway Condition With Significant Anesthesia Implications and the Importance of Preoperative Diagnosis

**DOI:** 10.7759/cureus.88326

**Published:** 2025-07-19

**Authors:** Odai Khamash, Shoaib Nawaz, Roni Mendonca, Ejaz Khan

**Affiliations:** 1 Anesthesiology, New York Medical College, Metropolitan Hospital Center, New York, USA

**Keywords:** airway anomaly, anesthesia implications, preoperative diagnosis, tracheal perforation, tracheocele

## Abstract

Tracheocele, a rare airway condition involving abnormal dilation of the tracheal wall, poses rare but crucial challenges in anesthesia management and carries the potential for serious complications if not promptly identified preoperatively. Our case highlights the importance of early diagnosis and tailored anesthetic planning in a patient with a large tracheocele undergoing colorectal resection surgery. The surgery was successfully performed using a combined spinal-epidural (CSE) anesthesia with moderate sedation, avoiding the potential risks associated with general anesthesia and positive pressure ventilation in this compromised airway scenario.

## Introduction

Tracheocele is a rare condition characterized by the out-pouching of the tracheal mucosa through a weak point in the tracheal wall, forming an air- or fluid-filled cystic lesion [1]. These diverticula are typically located along the right posterolateral wall of the trachea, most commonly at the junction between the cartilaginous and membranous portions due to the absence of supportive esophageal structures on that side [2]. The condition may be congenital or acquired. Congenital tracheoceles are thought to arise from developmental defects of the tracheal cartilage or musculature and are less common than acquired forms [1]. Acquired tracheoceles are usually secondary to prolonged increased intraluminal pressure and structural weakness of the tracheal wall, as seen in patients with chronic cough, chronic obstructive pulmonary disease (COPD), or repeated respiratory infections.

The incidence of tracheocele is not well documented given its rarity and the fact that it is often discovered incidentally on imaging. Nonetheless, several case reports have described its presence, and Rokitansky first reported this tracheal lesion in a postmortem study in 1846 [3,4]. Most patients are asymptomatic, though some may present with cough, recurrent respiratory infections, or airway obstruction symptoms.

Tracheocele holds particular clinical relevance in anesthesia due to the potential for serious complications during airway management. These include difficult intubation, ventilation through a false lumen, accidental rupture or tracheal injury, pneumomediastinum, and subcutaneous emphysema [5-9]. Consequently, preoperative identification and planning are crucial to minimize risk.

In this case report, we present a 72-year-old woman with a large, incidentally discovered tracheocele scheduled for colorectal resection. The report emphasizes anesthetic decision-making in the context of compromised airway anatomy and highlights the importance of multidisciplinary planning, including standby general anesthesia, extracorporeal membrane oxygenation (ECMO), and cardiothoracic consultation, to ensure patient safety in high-risk airway scenarios. We also review the current literature and outline perioperative anesthetic strategies used in this rare but significant condition.

## Case presentation

**Figure 1:**
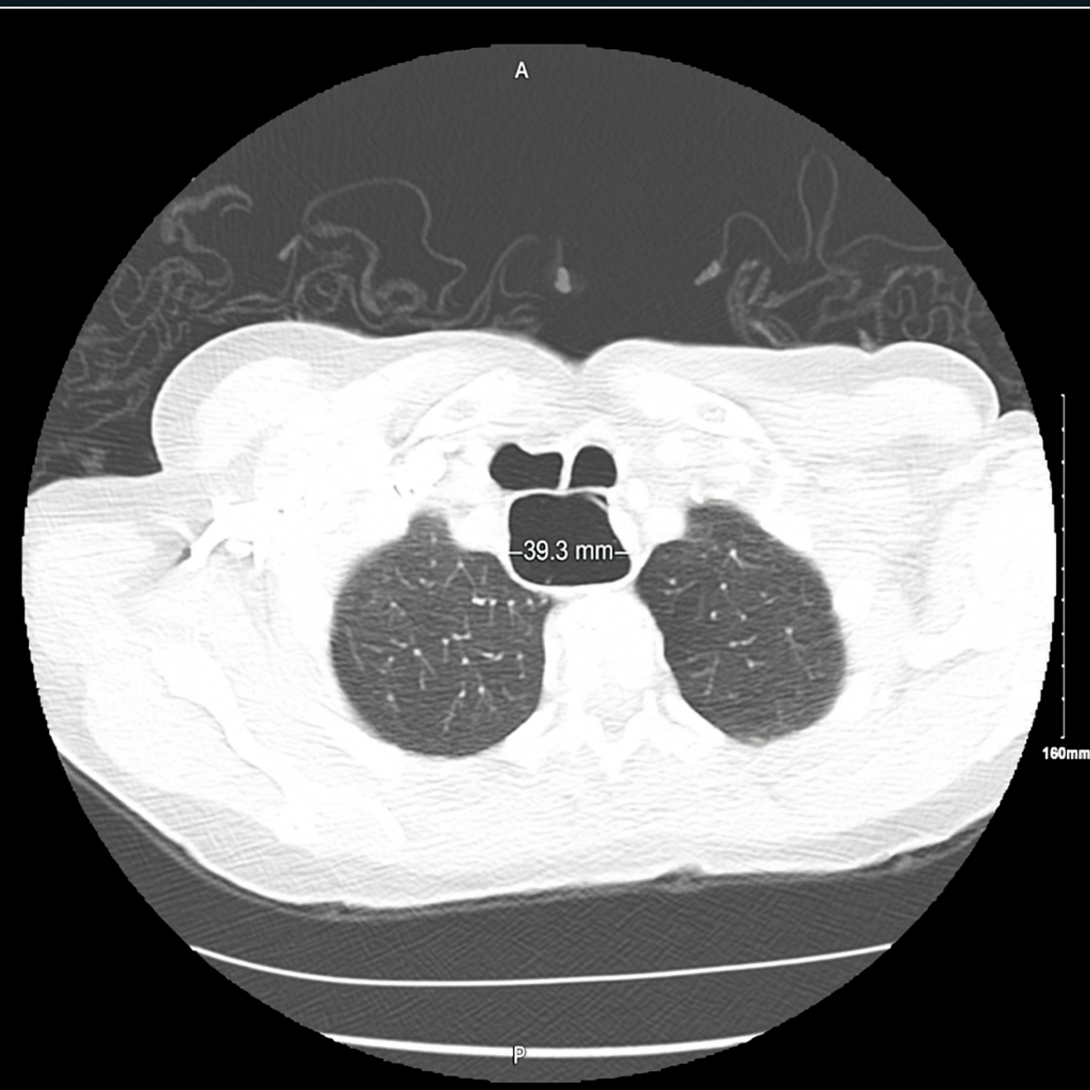
CT scan displaying a 39.3 mm tracheocele along the right lateral tracheal wall, indicative of significant airway dilatation.

**Figure 2:**
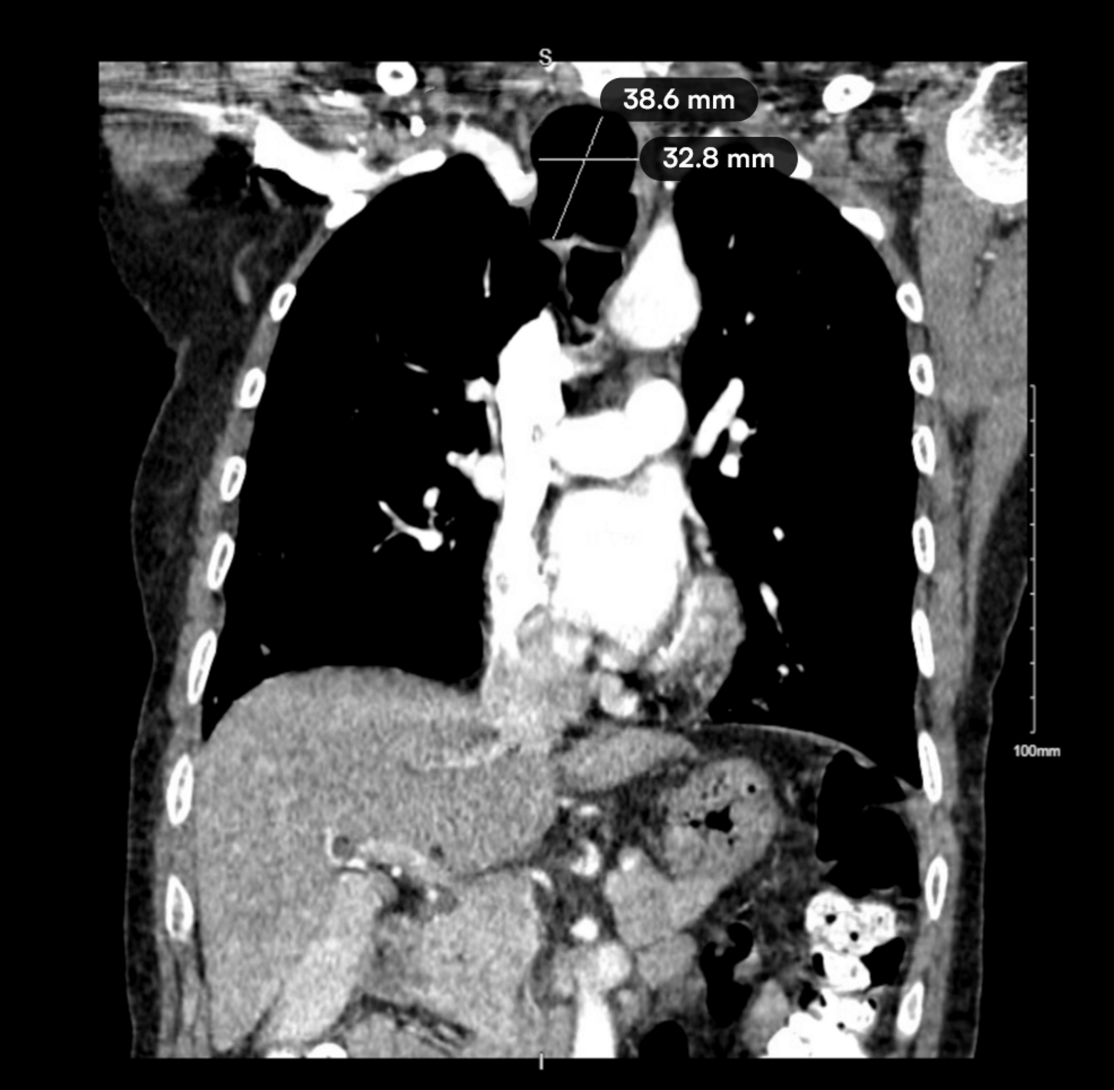
CT scan displaying a 38.6x32.8 mm tracheocele along the tracheal wall

A 72-year-old female with a history of severe bronchiectasis and chronic cough presented with recurrent per-rectal bleeding. Initial workup revealed microcytic anemia, and sigmoidoscopy showed a fungating rectal mass. She was diagnosed with rectal adenocarcinoma and scheduled for colorectal resection. Preoperative imaging, including thoracoabdominal CT scan, incidentally revealed multiple large pocket-like tracheocele extending from the tracheal inlet down below the carina, measuring 39 × 33 mm at the widest point (Figures 1, 2).

Given the presence of multiple tracheoceles extending along the tracheal wall and below the carina, and the anticipated difficulty in safely positioning the endotracheal tube (ETT) cuff beneath the defects, as well as the urgency of the surgical intervention, a multidisciplinary planning meeting was held involving the Anesthesia, General Surgery, Cardiothoracic Surgery, and ICU teams. To enhance preparedness and ensure patient safety, the team decided to proceed with regional anesthesia using a combined spinal-epidural (CSE) technique. General anesthesia, including the availability of ECMO, was kept as a contingency plan in the event of airway compromise, ventilation failure, or circulatory collapse, with equipment and personnel placed on standby during the procedure. The procedure was successfully carried out under CSE anesthesia with moderate sedation maintained via a propofol infusion.

CSE was achieved with 1.6 ml of 0.75% hyperbaric bupivacaine and 20 mcg of fentanyl, and an epidural catheter was inserted at the L2-L3 level. Ropivacaine 0.2% infusion (5 ml/hr) was used through the epidural catheter. A laparoscopic-assisted (gas-less) inter-sphincteric abdominoperineal resection was done. The patient was kept on spontaneous breathing while receiving 5 liters of oxygen through a face mask. She remained hemodynamically stable, with oxygen saturation levels exceeding 94%, and did not require any vasopressors.

The surgery was successfully completed without any complications. The patient was then transferred to the Surgical Intensive Care Unit for postoperative monitoring, where she remained stable and was discharged home after five days, with no additional complications.

The patient was referred to cardiothoracic surgery. A reassessment with follow-up imaging was conducted, confirming the presence of large tracheoceles. The option of surgical intervention including tracheal stenting was offered, but the patient declined intervention. She was followed up at three and six months postoperatively and remained stable without respiratory symptoms or new complications.

## Discussion

A tracheocele is an abnormal growth of tracheal tissue and presents as a para-tracheal air cyst. It can be either congenital or acquired and may present as single or multiple cysts [10]. The trachealis muscle has been described in Miller’s anatomy as having transverse bands with some weak amuscular areas from which tracheoceles can generate.

Patients typically present with symptoms such as chronic cough, stridor, or recurrent respiratory infections [1]. They may also experience dysphagia, dyspnea, and dysphonia, which can result from local mass effects or vocal fold paralysis due to compression of the recurrent laryngeal nerve [1]. The differential diagnosis includes conditions such as laryngoceles, pharyngoceles, Zenker diverticulum, pulmonary apical hernias, blebs, pneumomediastinum, and bullae [11].

While there is no standardized cure for tracheocele, treatment is usually conservative for asymptomatic cases or elderly patients including antibiotics, mucolytic agents, and physiotherapy. The age of the patient, the clinical presentation, and the presence of comorbidities should be taken into account when choosing a treatment approach in symptomatic patients. Surgical or endoscopic interventions may be considered depending on severity and complications [12]. 

Kapoor et al. noted that positive pressure ventilation can lead to serious complications and emphasized that intraoperative airway management should involve spontaneous ventilation, bronchoscopy for intubation, and positioning the ETT cuff distal to the defect [12]. However, our patient presented a particularly complex challenge. Imaging revealed multiple large tracheoceles, with pocket-like extensions along the tracheal wall and a notably thin wall separating them from the tracheal lumen. Critically, these defects extended beyond the carina, creating a high-risk scenario for airway instrumentation. In our case, we thought that even advanced airway techniques such as awake fiberoptic intubation or video-laryngoscopy offer limited advantages, as the irregular anatomy and distal location of the defects make safe and accurate positioning of the ETT extremely difficult. The risk of misplacement, tracheal injury, or rupture of the tracheocele during intubation or positive pressure ventilation was deemed unacceptably high.

Given these concerns and the urgent need for surgical intervention, the decision was made to proceed with regional anesthesia using a CSE technique. As a contingency plan, general anesthesia was kept on standby, with preparations including fiberoptic bronchoscopy, a double-lumen tube (DLT), and ECMO. Both equipment and trained personnel were readily available to enable an immediate transition to airway management and cardiopulmonary support should neuraxial anesthesia prove insufficient or if intraoperative complications occurred.

This case underscores the importance of individualized anesthetic planning in patients with complex airway anatomy. It also highlights the value of early multidisciplinary collaboration in optimizing patient safety and outcomes in high-risk scenarios.

Previous studies and case reports mainly highlight the complications related to tracheal intubation and positive pressure ventilation. For instance, Moller et al. reported an accidental perforation of a tracheocele due to endotracheal intubation, which led to postoperative pneumomediastinum [6]. Consequently, it is essential to conduct vigilant postoperative airway monitoring, paying close attention to symptoms such as dyspnea, wheezing, stridor, grunting, and air hunger. These symptoms serve as critical indicators of potential tracheocele-related complications, including airway obstruction or rupture, as some airway issues may arise after extubation.

If general anesthesia is considered necessary, the literature suggests the use of fiberoptic bronchoscopy and/or video laryngoscopy, along with appropriate placement of the ETT cuff and careful lung ventilation [13,14].

Despite the successful outcome in this case, several limitations warrant acknowledgment. First, long-term follow-up data beyond six months are lacking, which limits our ability to assess the progression of the tracheocele or any delayed complications. Second, the rarity of this condition inherently limits generalizability; while our management approach was effective here, it may not be applicable to all cases. Third, certain technical constraints-such as limited availability of advanced imaging, may affect the reproducibility of this strategy in other settings. Nonetheless, our experience emphasizes the importance of early diagnosis, multidisciplinary planning, and a flexible anesthetic approach tailored to individual anatomy and clinical context. 

## Conclusions

This case highlights the critical importance of early recognition and individualized anesthetic planning in managing patients with tracheocele, a rare but potentially high-risk airway anomaly. The successful use of neuraxial anesthesia may be an effective strategy to minimize airway trauma and avoid the risks associated with positive pressure ventilation. High-resolution preoperative imaging, such as CT scans of the neck and chest, helped to accurately assess the size and location of the tracheocele. Early multidisciplinary coordination, including anesthesiology, surgical, thoracic, and ECMO teams, can be critical for optimal preparedness. Given the rarity of this condition, heightened clinical awareness and thorough preoperative evaluation are essential to ensuring safe and successful perioperative outcomes.

## References

[1] Kallel S, Chaabouni MA, Thabet W, Mnejja M, Ben Mahfoudh K, Charfeddine I (2022). Tracheocele: a rare entity. Iran J Otorhinolaryngol.

[2] Martinez SA, Ruiz-Mojica CA, Rivera-Rivera G, Perez-Ortiz G, Morrero JP, Lopez PP (2023). Incidental tracheocele as an unusual presentation of pneumomediastinum in the trauma setting. Am J Case Rep.

[3] Ramadass T, Rayappa C, Santosham R, Krishna Prasad PN, Rao P (2015). Tracheocele presenting with intermittent dysphonia: a case report. Indian J Otolaryngol Head Neck Surg.

[4] Rokitansky K (1846). Handbuch Der Pathologischen Anatomie: Handbuch Der Allgemeinen Pathologischen Anatomie, Volume 1.

[5] Hutton M, Brull R, Macfarlane AJ (2018). Regional anaesthesia and outcomes. BJA Educ.

[6] Möller GM, ten Berge EJ, Stassen CM (1994). Tracheocele: a rare cause of difficult endotracheal intubation and subsequent pneumomediastinum. Eur Respir J.

[7] Dohlman LE, Kwikiriza A, Ehie O (2020). Benefits and barriers to increasing regional anesthesia in resource-limited settings. Local Reg Anesth.

[8] Almeida CR, Vieira LS, Cunha P, Gomes A (2022). Low-dose spinal block combined with epidural volume extension in a high-risk cardiac patient: a case-based systematic literature review. Saudi J Anaesth.

[9] Kurt A, Sayit AT, Ipek A, Tatar IG (2013). A multi detector computed tomography survey of tracheal diverticulum. Eurasian J Med.

[10] Botti C, Maccarrone F, Bernardelli G, Lupi M, Presutti L, Mattioli F (2020). Siblings with acquired tracheocele: possible hereditary etiopathogenesis?. Head Neck Pathol.

[11] Toscano L, Terra D, Salisbury S, Arechavaleta N (2019). Surgical resection of tracheal diverticulum with haemoptysis as unusual presentation. Case Rep Surg.

[12] Tanrivermis Sayit A, Elmali M, Saglam D, Celenk C (2016). The diseases of airway-tracheal diverticulum: a review of the literature. J Thorac Dis.

[13] Kapoor R, Truong A, Truong DT (2021). Tracheocele showing a distinctive bullfrog-breathing pattern. Anesthesiology.

[14] Mitsuzawa K, Kumagai T, Uchida H, Shimizu T (2023). Positional relationships between a tracheal diverticulum and the tracheal tube under general anesthesia: a single-center observational and simulation study. BMC Anesthesiol.

